# Combined Retroperitoneal and Transperitoneal Laparoscopic Procedures by a Single Surgeon: Boon to Economically and Medically Backward Areas

**DOI:** 10.7759/cureus.13152

**Published:** 2021-02-05

**Authors:** Jayanta Kumar Das, Mayank Singh, Gordon Rangad

**Affiliations:** 1 Department of General and Minimally Invasive Surgery, Nazareth Hospital, Shillong, IND

**Keywords:** combined laparoscopic procedures, retroperitoneal laparoscopic urological surgery, urolithiasis

## Abstract

Introduction

This study was done to evaluate our experience of combining a retroperitoneal laparoscopic urological operation with other transperitoneal laparoscopic operations.

Materials and methods

We present a retrospective study of a series of 20 cases of retroperitoneal laparoscopic urological surgeries combined with at least one transperitoneal laparoscopic procedures, performed by a senior minimally invasive surgeon, between March 2013 and August 2020. We have excluded three patients where either of the procedures required conversion to open surgery. We retrospectively reviewed all the data regarding the patient’s demographics, combined surgical procedures done, operative time taken, blood loss, intraoperative and postoperative complications, and days of hospital stay.

Results

Total of 20 patients had undergone simultaneous retroperitoneal and transperitoneal laparoscopic procedures. A total of nine (45%) cases comprised a combination of retroperitoneal laparoscopic ureterolithotomy and laparoscopic cholecystectomy. Two patients had undergone a combination of three laparoscopic procedures in the same operation. The mean hospital stay was 3.6 days. Blood loss was minimal to moderate in all the patients, none needed any perioperative blood transfusion. No major complications were noted in any patients.

Conclusion

Combining a retroperitoneal laparoscopic urological procedure with another transperitoneal laparoscopic surgery is very much feasible. It becomes even easier and relevant provided both the procedures are performed by a single, experienced laparoscopic surgeon.

## Introduction

The first laparoscopic urological procedure was reported by Dr William Schuessler and his colleagues [1]. Since then, laparoscopic surgery in urology has gradually become a standard, safe, and effective armamentarium [2]. It has emerged as an advanced tool for the newer generation of urologists to treat various urological diseases [3].

With the advances in the minimally invasive surgery, various laparoscopic procedures are being done simultaneously for treating co-existent abdominal pathology together at one operation. Simultaneous laparoscopic procedures for general surgical diseases have received good acceptance [3-6]. Many isolated case reports and case series of concomitant urological procedures or with other laparoscopic operations also had been documented [2,3,6-10]. Though some authors have described a few cases, not much literature is available on the combination of retroperitoneal laparoscopic procedure with another transperitoneal laparoscopic procedure [3,9,10]. Here we have made an attempt to elaborate the cases that had undergone simultaneous laparoscopic retroperitoneal urological procedure and other laparoscopic transperitoneal procedure together at a single operation, done at our institute.

There are multiple advantages of concomitant surgeries that include operation under single anesthesia, single and shorter hospital stay, decreased amount of medication and cost of two interventions, and, very importantly, reduced psychological stress for the patients [2-4,6,11].

Urolithiasis constitutes a bulk of surgical cases in our state. Our center does not have a specialized Department of Urology. Our state has a limited number of urologists, who are overburdened with patients. We, the general surgeons, therefore, have been routinely performing urological surgeries. Our center has been a pioneer in this region and has been doing laparoscopic urological surgeries since the beginning of this decade to provide the benefit of minimally invasive surgery over open surgery to our patients.

## Materials and methods

Herein we present our experience of 20 cases of combined retroperitoneal laparoscopic urological procedure for urolithiasis, with at least one transperitoneal laparoscopic procedures. We have excluded three patients who required conversion of either of the procedures to open surgery. All these cases were done at our center between March 2013 and August 2020 by a single experienced senior laparoscopic surgeon.

All patients had undergone preoperative ultrasonography and Intravenous Urogram (IVU) to diagnose the pathology, to see the function of both the kidneys, and to see the approximate size and position of the stones. Whenever indicated and the patient could afford, a contrast-enhanced computed tomography (CECT) scan of the urinary tract was performed. In all the cases except the nephrectomy, a plain picture kidney, ureter and bladder (KUB) was done on the day of operation.

**Figure 1:**
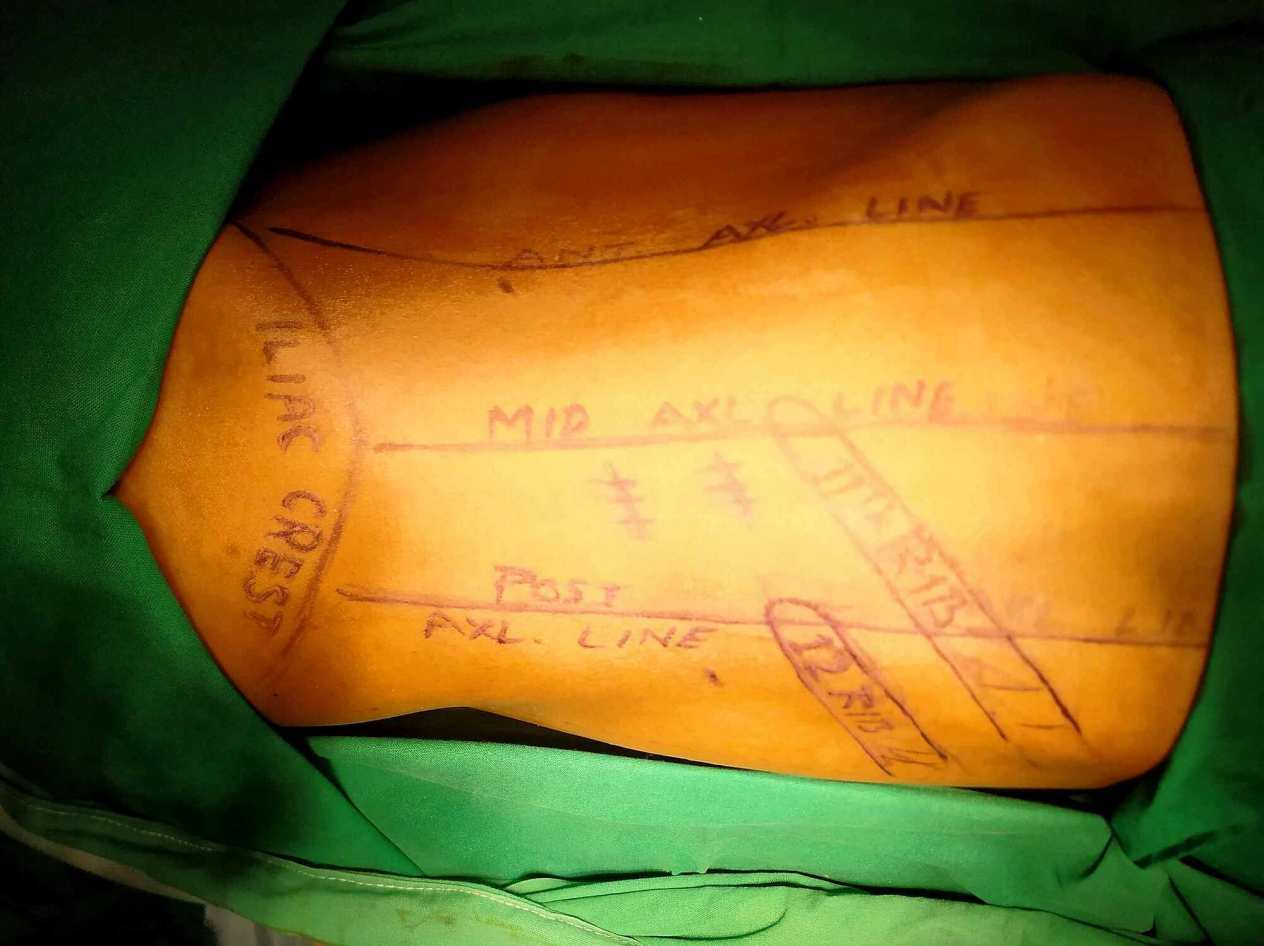
Port positions in retroperitoneal laparoscopic operations in relation to the ribs.

We used routinely a 12 mm camera port either two fingers below the tip of 12th rib or at the tip of 12th rib along its long axis, where we expected some difficulty in the operation that may require conversion to open. Therefore, if the need for conversion arises, we could extend the incision along the 12th rib axis. Two other 5 mm ports were used in the standard positions in ‘I’ configuration in all the cases (Figure 1). Till now we have not got any case where we had to put any extra ports. We routinely keep a double J stent in all cases of laparoscopic ureterolithotomies and pyelolithotomies, which were removed after 8-12 weeks. We routinely catheterize the patient immediately after the operation. Usually, drain and catheter were removed on the third postoperative day, unless the drain was greater than 30-50 ml.

**Figure 2:**
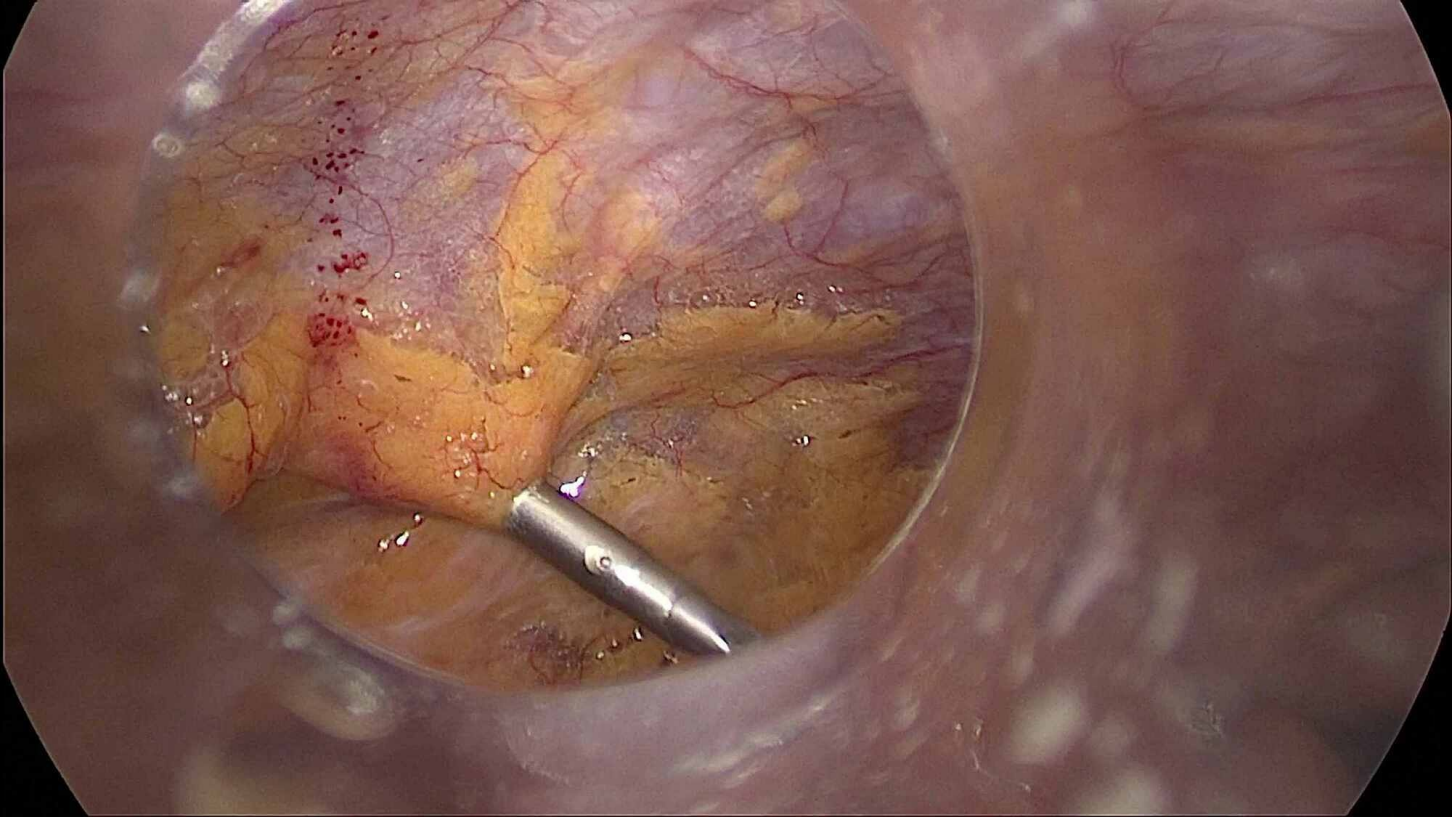
Gall bladder fundus retracting port inserted through the same port site of laparoscopic pyelolithotomy.

In all the patients who had undergone a combination of right-sided retroperitoneal operation with laparoscopic cholecystectomy (LC), we first performed the retroperitoneal operation. Therefore, we could use the anterior 5 mm port in the right lower abdomen as the gall bladder fundus retraction port during subsequent LC, thereby reducing the number of ports (Figure 2). In these cases, we kept the retroperitoneal drain through the posterior 5 mm port. Otherwise, in all other patients, we kept the drain through the anterior 5 mm port. Other patients had undergone the easier transperitoneal operation first followed by the retroperitoneal urological procedures.

Two patients who had undergone combined retroperitoneal right nephrectomy with LC were patients of non-functioning kidney due to long-standing renal pelvic stone. In these patients, the loss of renal function was confirmed by CECT-IVU and by measuring the cortical thickness and vascularity on Doppler ultrasonography of the kidney. Renal scan is not available in our state. The kidney was removed after a little extension of the 12 mm port wound.

Patients' data were retrospectively analyzed with respect to the demographics, procedures performed, operative time taken, blood loss, any intraoperative and postoperative complications, duration of hospital stay and cost analysis. The operative time taken was defined as the time taken from the placement of the first incision to the completion of last skin clips, including the time taken for a change of position in between the operations.

Any blood loss of approximately less than 100 ml during the operation was categorized as minimal blood loss. If any patient required blood transfusion because of intraoperative blood loss, they were categorized as severe blood loss. Patients between these two groups were categorized as moderate blood loss.

## Results

A total of 297 numbers of successful laparoscopic urological operations were performed by the authors over a period of nine and a half years from March 2011 to September 2020. Out of these, a total of 20 cases of retroperitoneal urological procedures were performed in combination with at least one transperitoneal procedure (Table 1). The first case of combined surgeries was performed in May 2013 and the last in August 2020. Eleven (55%) were females and nine (45%) were male patients. Age of the patients ranged from 20 to 81 years.

**Table 1:** Detailed finding of our study. Rt - Right; RLU - Retroperitoneal laparoscopic ureterolithotomy; LC - Laparoscopic cholecystectomy; S - shifting time; Lt - Left; TPU - Transperitoneal laparoscopic ureterolithotomy; RLP - Retroperitoneal laparoscopic pyelolithotomy; FC - Fimbrial cystectomy; RLN - Retroperitoneal laparoscopic nephrectomy; LA - Laparoscopic appendicectomy; URSL - Ureteroscopic lithotripsy; LH - Laparocopic hernia repair; m - Mini; Lap. - Laprascopic; POD - Postoperative day; TAPP - Transabdominal preperitoneal

Serial Number	Operations Done	Total Cases	Age/ Sex	Time Taken	Blood Loss	Discharged On
1	Lap. Cholecystectomy and Lap. Retroperitoneal Ureterolithotomy	9	33/F	2:40hr (Total)- 1:05 hr (Rt RLU)+ 1:17hr (LC)+ 0:18hr (S)	Moderate	3^rd^ POD
			29/F	3:00hr (Total)- 1:05 hr (Rt RLU)+ 1:38hr (LC)+ 0:17hr (S)	Moderate	3^rd^ POD
			63/M	1:45hr (Total)- 0:55 hr (LC)+ 0:38hr (Lt RLU)+ 0:12hr(S)	Minimal	4^th^ POD
			27/F	2:04hr (Total)- 0:43 hr (LC)+ 1:09hr(Lt RLU)+ 0:12hr (S)	Minimal	3^rd^ POD
			20/F	1:40hr (Total)- 0:45 hr (LC)+ 0:40hr (Lt RLU)+ 0:15hr (S)	Minimal	7^th^ POD
			37/M	1:39hr(Total)- 0:39 hr (LC)+ 0:48hr (Lt RLU)+ 0:12hr (S)	Minimal	3^rd^ POD
			29/F	1:50hr (Total)- 0:46 hr (LC)+ 0:50hr (Lt RLU)+ 0:14hr (S)	Minimal	3^rd^ POD
			41/M	1:28hr (Total)- 0:42 hr (Rt RLU)+ 0:29hr (LC)+ 0:17hr (S)	Minimal	3^rd^ POD
			45/M	1:53hr (Total)- 0:48 hr (LC)+ 0:52hr (Lt RLU)+ 0:13hr (S)	Minimal	3^rd^ POD
2	Lap. Cholecystectomy and bilateral Lap. Ureterolithotomy	1	39/F	2:33hr (Total)- 1:43 hr (LC & Rt TPU)+ 0:34hr (Lt RLU)+ 0:16hr (S)	Minimal	3^rd^ POD
3	Lap. Cholecystectomy and Lap. Pyelolithotomy	2	43/F	2:16hr (Total)- 0:46 hr (LC)+ 1:16hr (Lt RLP)+ 0:14hr(S)	Moderate	3^rd^ POD
			49/F	1:55hr (Total)- 0:38 hr (LC)+ 1:02hr (Lt RLP)+ 0:15hr (S)	Minimal	3^rd^ POD
4	Lap. Cholecystectomy, Lap. Pyelolithotomy and Fimbrial Cystectomy	1	81/F	2:55hr (Total)- 1:13 hr (Rt RLP)+ 1:27hr (LC & Rt FC)+ 0:15 hr (S)	Moderate	3^rd^ POD
5	Lap. Cholecystectomy and Lap. Nephrectomy	2	57/F	2:39hr (Total)- 1:44 hr (Rt RLN)+ 0:40hr (LC)+ 0:15hr (S)	Moderate	7^th^ POD
			68/M	2:01hr (Total)-1:56 hr (Rt RLN)+ 0:48hr (LC)+ 0:17hr(S)	Minimal	3^rd^ POD
6	Lap. Appendicectomy and Lap. Retroperitoneal Ureterolithotomy	1	28/M	1:45 Hr(Total)-0:37 hr (LA)+ 0:56hr (Lt RLU)+ 0:12hr (S)	Minimal	4^th^ POD
7	Lap. Cholecystectomy, URSL and Lap. Retroperitoneal Ureterolithotomy	1	57/M	2:12hr (Total)- 0:45 hr (Rt RLU)+ 0:49 hr (LC)+ 0:23hr (Lt URSL)+ 0:15hr (S)	Minimal	4^th^ POD
8	Lap. TAPP repair of Inguinal Hernia and Lap. trans-peritoneal Ureterolithotomy	1	36/M	2:10hr (Total)- 1:05 hr (Rt LH)+ 0:47hr (Lt RLU)+ 0:18hr (S)	Minimal	3^rd^ POD
9	Lap. B/L Ureterolithotomy (Left- Retroperitoneal and Right- Transperitoneal)	1	37/M	2:08hr (Total)- 0:48 hr (Lt RLU)+ 1:05hr (Rt TPU) + 0:15hr (S)	Minimal	3^rd ^POD
10	Mini Lap. Cholecystectomy and Mini Lap. Retoperitoneal Ureterolithotomy	1	46/F	2:11hr (Total)- 0:54 hr (m LC)+ 1:00hr (Lt m RLU) + 0:17hr (S)	Minimal	4^th^ POD

**Figure 3:**
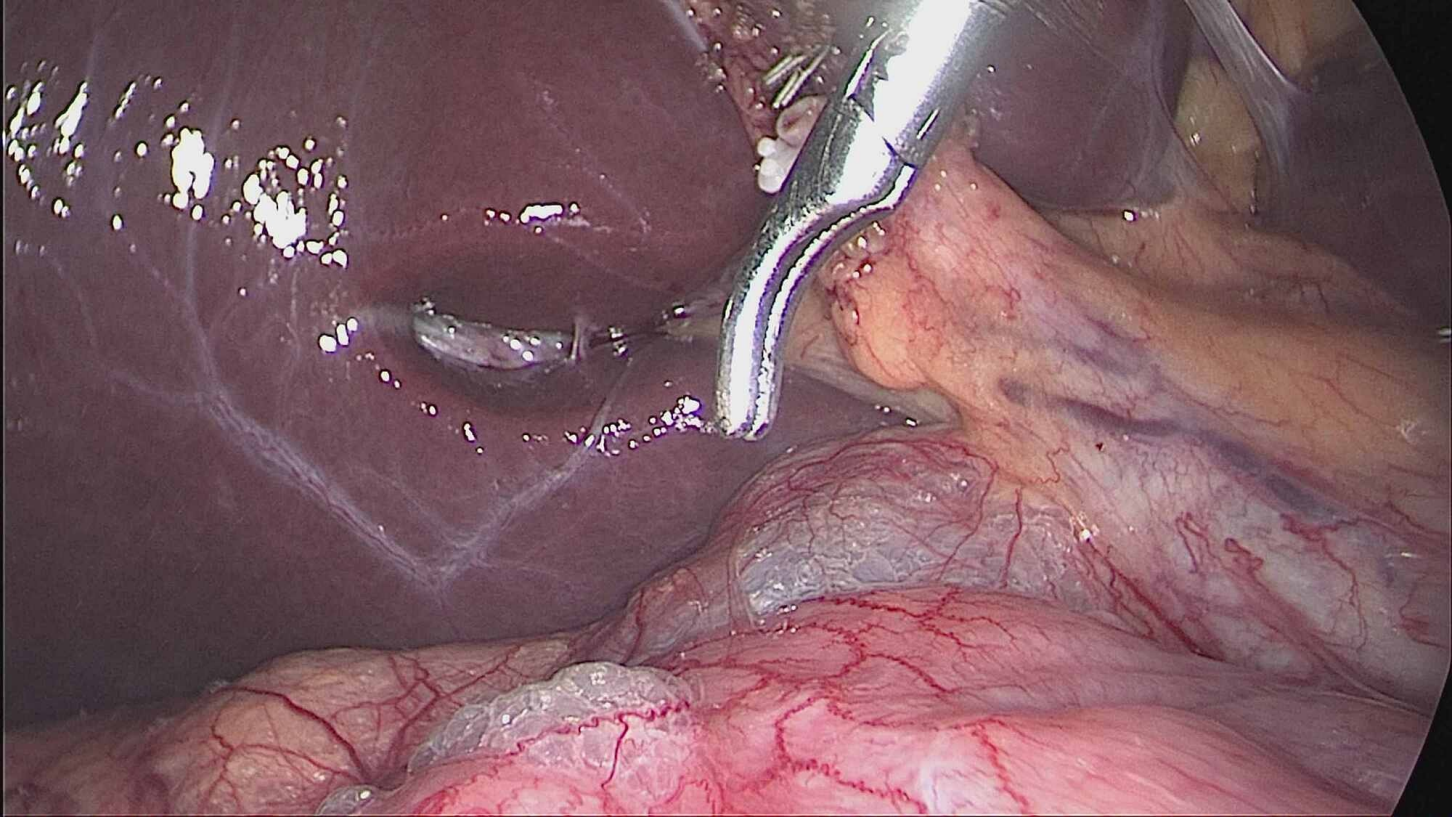
Laparoscopic cholecystectomy after retroperitoneal laparoscopic ureterolithotomy: gas bubbles seen in the retroperitoneum.

**Figure 4:**
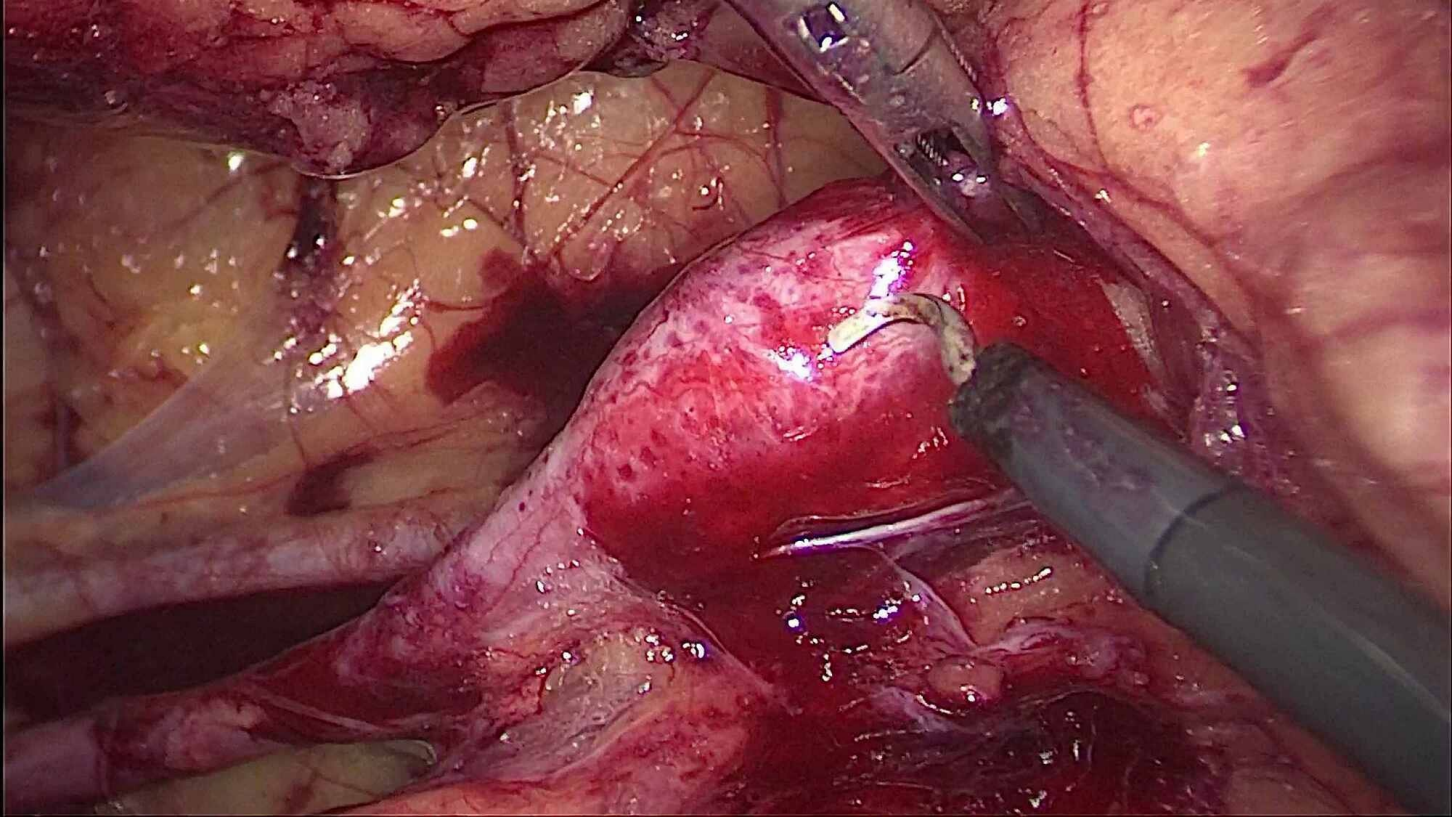
Mini laparoscopic ureterolithotomy with 3 mm instruments.

Out of the total 20 cases, the most common operation (45%) were a combination of retroperitoneal laparoscopic ureterolithotomy (RLU) with LC (Figure 3). Another patient had undergone mini laparoscopic cholecystectomy and retroperitoneal mini laparoscopic ureterolithotomy (Figure 4).

**Figure 5:**
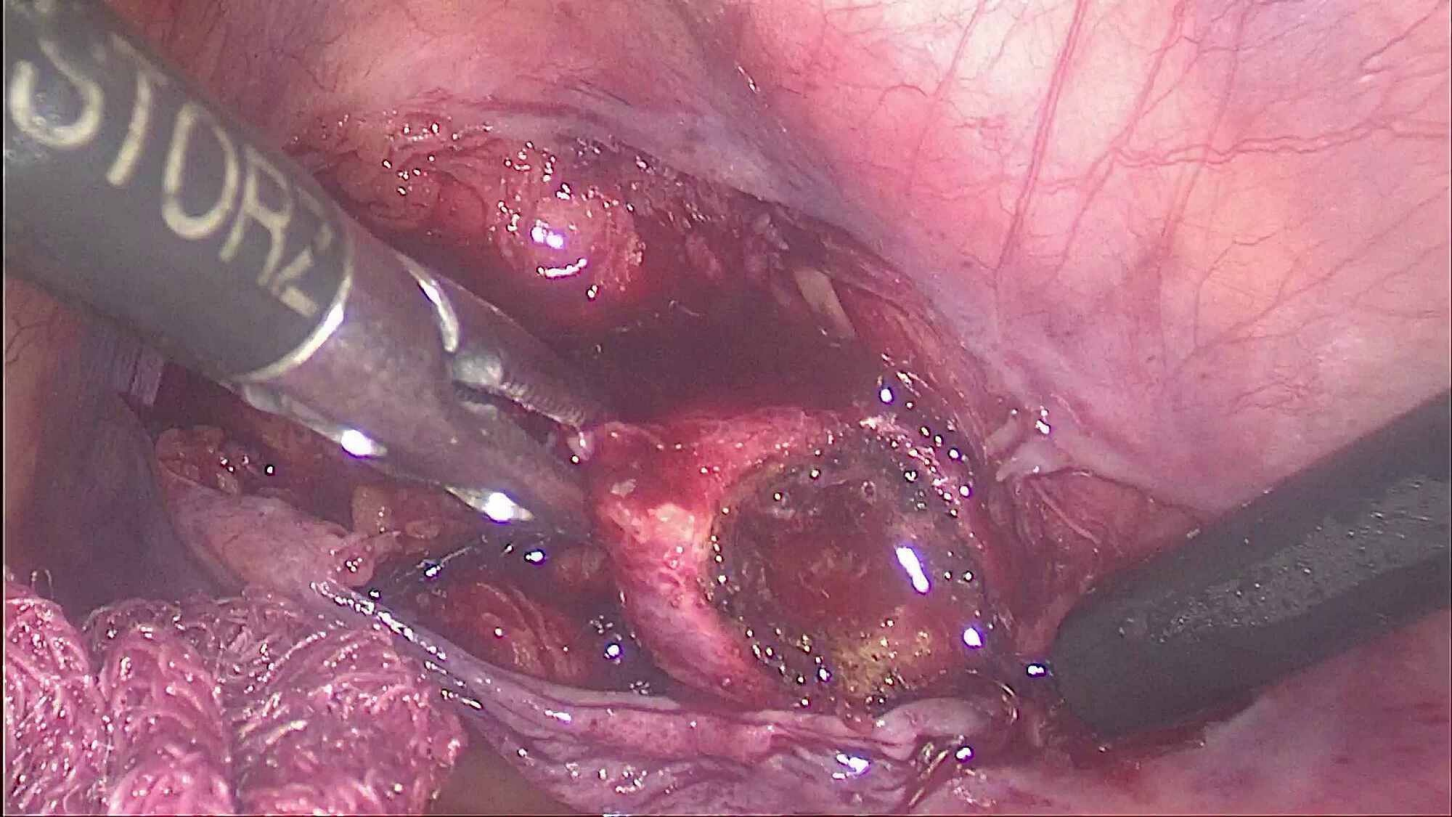
Transperitoneal laparoscopic ureterolithotomy: extraction of the stone.

Two of our patients had undergone a combination of one retroperitoneal and two transperitoneal procedures during the same operation. One of them had undergone a combination of LC and bilateral laparoscopic ureterolithotomy. She had a right lower and a left upper ureteric stones along with chronic calculous cholecystitis. She underwent transperitoneal ureterolithotomy (TPU) on the right side and RLU on the left side, along with LC (Figure 5). The other patient had undergone a combination of retroperitoneal laparoscopic pyelolithotomy (RLP) on the right side and transperitoneal excision of a large right fimbrial cyst and LC. Another patient had undergone a combination of RLU on the right side and LC with another endoscopic urological procedure (left-sided ureteroscopic lithotripsy).

Our mean operative time was 2.08 hours (range from 1.28 to 3.00 hours). Both the shortest and longest operating times were taken for patients who had undergone RLU with LC. We required 12-18 minutes (mean 14.95 minutes) for the change of the patient’s position in between the procedures.

**Figure 6:**
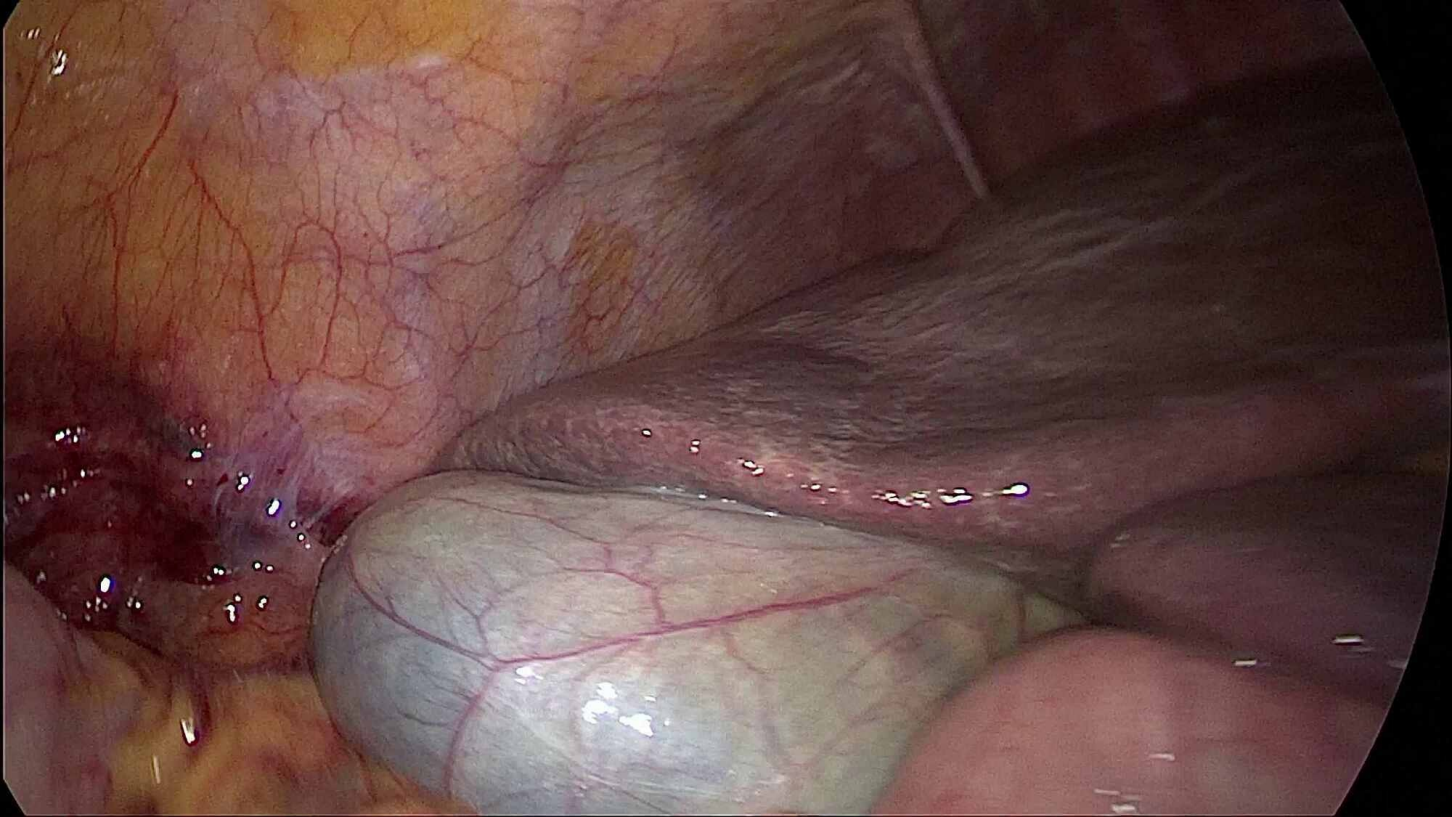
Laparoscopic cholecystectomy after retroperitoneal laparoscopic nephrectomy: bloodstains seen in the retroperitoneum.

Blood loss in all the patients was minimal to moderate and no patients required blood transfusion due to intraoperative blood loss. None of our patients developed any major postoperative complications. Two patients had minor port site wound infection. Two patients, one each of combined LC with RLU and nephrectomy (Figure 6), had prolonged drainage for seven days.

The mean hospital stay for these patients was 3.6 days (range between three to seven days). Fourteen (70%) patients were discharged on the third postoperative day after the removal of drain, catheter, and skin clips.

## Discussion

The first laparoscopic urological procedure was reported by Dr William Schuessler et al. [1]. They presented 12 cases of endoscopic pelvic lymphadenectomy in the *Journal of Urology* in the year 1991. Since then, gradually, laparoscopy has become an important part in the management of urological diseases [2,3]. Due to the availability of other minimally invasive procedures like laser lithotripsy, extracorporeal shock wave lithotripsy (ESWL), and retrograde intrarenal surgery (RIRS), the use of laparoscopic surgery in the management of urolithiasis is still negligible. But in few exceptions, its use has been extensively described in the literature [12-14].

Patients undergoing laparoscopic surgeries have the advantages of early postoperative recovery, better cosmesis, and shorter hospital stay. Therefore, surgeons have sought to extend this benefit to combined laparoscopic procedures for the treatment of two or more diseases [2,3,6]. This provides patients with the benefit of exposure to single anesthesia, reduced hospitalization with single recovery period, decreased use of perioperative medications, decreased morbidity, including decreased psychological stress, and overall better cost-effectiveness [2-4,6,15]. In this part of India, one of the economically and medically backward areas, a bulk of surgical patients comprises of urolithiasis. With the lack of a sufficient number of fully equipped urology centers in the state, our center has been using laparoscopic surgery as a safe, effective, and better alternative than open surgery, especially for stone diseases.

Most authors have found the combination of surgeries to be very cost-effective [3,4,6,11,15]. Cartapatti M and his colleagues commented that in countries such as Brazil, where institution’s financial problems may interfere with treatment selection, a combination of such procedures for synchronous pathology might be a good selection [2]. Maurya K and his colleagues, in a case series of a larger number of 64 concomitant laparoscopic surgeries, also opined that a concomitant laparoscopic urological procedure reduces the cost and morbidity of the patients [3]. In our study, we have found that the average total cost of combined procedures decreases the cost of operation than if the patient had undergone operations for each pathology separately. The cost decreases due to the decreased number of hospital days with a single break from work, decreased use of IV fluids and medications, instrumentation costs, as well as cost of attendants for the patients. In economically poor areas such as ours, where patient’s financial problems may interfere with treatment selection, combining together such procedures in a single surgery might be a good answer.

Another important aspect of the combination of laparoscopic procedures is to use fewer ports and the same access sites for both procedures adding at maximum one to two extra ports [2,15,16]. But this is better feasible in combined transperitoneal laparoscopic operations. In our series, only in those patients who had undergone the combination of LC with a right-sided retroperitoneal laparoscopic operation, we could use the right lower abdominal port as a common one.

Combined laparoscopic surgery may have some disadvantages in comparison to combined open surgery. Hemodynamic changes secondary to prolonged pneumoperitoneum could become a limitation to this approach [2,3,8]. Meininger D and his colleagues, in a prospective study, evaluated hemodynamic parameters in patients on Trendelenberg position with pneumoperitoneum pressure set at 12 mm of Hg during laparoscopic radical prostatectomy [17]. They demonstrated that the head-down position causes only a significant increase in central venous pressure, while the induction of pneumoperitoneum for a period of four hours significantly affected the mean arterial pressure. All other hemodynamic parameters nearly remained unaffected. Luo CF and his colleagues in their study had concluded that a prolonged pneumoperitoneum of four hours results in decreased splanchnic blood flow and increased oxidative stress both during pneumoperitoneum and sometime after deflation [18]. The clinical relevance of oxidative stress after pneumoperitoneum is related to hepatic, lung, and renal injuries. All these reported changes were transient, with no permanent impairment in patient’s renal and cardiopulmonary functions, but need further investigation. Meininger et al. opined that prolonged laparoscopic surgeries therefore be avoided in patients with cardiopulmonary morbidities [17]. None of our patients had any intraoperative hemodynamic instability

Many authors were concerned of combining two or more procedures in a single surgery as it may prolong the operating time, increase the chance of blood loss and complications, and increased hospital stay [2-4,8,15,19]. Tsivian A and his colleagues, in 2009, described 19 patients who underwent concomitant laparoscopic kidney surgery and cholecystectomy and reported an acceptable duration of surgery in addition to efficacy and safety of the procedure [7]. Similar findings were reported by Papilla R and his colleagues [15]. Wadhwa A and his colleagues, who published a paper on 130 combined laparoscopic or endoscopic procedures, also commented that simultaneous operations are feasible without significant addition in postoperative morbidity and hospital stay [6]. In our series, the operative duration for combined procedures was higher than the average time taken in any of the individual procedures, which is as per expectation. But this in no way affected the complication rate or morbidity. None of our patients required longer than three hours for completing their combined procedures.

Change of patient’s position in between may become another limitation in combined surgeries [10]. Papilia R and his colleagues in a series of 32 cases of simultaneously done laparoscopic urological surgeries for malignant pathologies reported their mean repositioning time to be of six minutes (range 5-10 minutes) [15]. They had performed all the operations by a transperitoneal approach. In our series, we had combined a retroperitoneal operation with transperitoneal operations. We needed to change the position of the patient from supine to lateral kidney position or vice versa and do re-cleaning and re-draping of the patients. We required between 12-18 minutes (mean 14.95 minutes), which is a little higher than them. But during this position changing, there was no pneumoperitoneum. Probably this repositioning time without any pneumoperitoneum also contributed to intraoperative hemodynamic stability of our patients.

Arnau ABM and his colleagues in a case series of four simultaneous colectomy and nephrectomy for malignant pathologies had opined that combined surgery in two different organs poses tactical difficulties. Also, a synchronous approach is greater biological aggression than performing two separate interventions. But this is possible especially when both the procedures are simple [10]. All our procedures were for nonmalignant simple pathology.

We had performed one combination surgery of left RLU and transabdominal preperitoneal (TAPP) repair of right inguinal hernia. This patient did not have any immediate post-operative as well as mesh-related infectious complicated over a follow-up period of about five years. Multiple studies have concluded that adding a clean-contaminated surgery to the inguinal or ventral hernia repair with mesh is not associated with an increased chance of mesh infection [20-22].

Very importantly, multiple studies have shown that a combination of laparoscopic surgeries in a single operation reduces the morbidity of anxiety and psychological stress of the patients with another admission and surgery [3,11]. All our patients were very happy when they were informed that we will perform the surgeries for all pathologies at the same operation under single anaesthesia.

## Conclusions

Combining a retroperitoneal laparoscopic urological procedure with another transperitoneal laparoscopic surgery is very much feasible. It even becomes easier and more relevant, provided both the procedures are performed by a single experienced laparoscopic surgeon.

This approach provides the benefit of minimally invasive surgery together with simultaneous management of coexisting pathologies to the patients and may probably be a good choice for economically and medically backward areas.

*Drawbacks of our study*: Most of our cases except three are a combination of laparoscopic cholecystectomy with another transperitoneal procedure. Though pain score and decreased requirement of postoperative analgesics is an important aspect of minimally invasive surgery, we could not comment on this aspect as this is a retrospective study and we had not collected any data regarding pain scores or had not used any protocolized analgesics.
